# Pregnant women’s experiences of the digital self-care program *women-in-motion* to manage physical activity and pelvic girdle pain: A qualitative study

**DOI:** 10.1177/20552076261459519

**Published:** 2026-06-09

**Authors:** Bodil Halvarsson, Annelie Gutke, Joanna Kvist, Jo Nijs, Mari Lundberg

**Affiliations:** 1 Department of Health and Rehabilitation, Unit of Physiotherapy, 195564 University of Gothenburg Institute of Neuroscience and Physiology, Sahlgrenska Academy, Gothenburg, Sweden; 2 Women in Motion Research Group, Department of Health and Rehabilitation, Unit of Physiotherapy, 195564 University of Gothenburg Institute of Neuroscience and Physiology, Sahlgrenska Academy, Gothenburg, Sweden; 3 Unit of Physiotherapy, 571308 Department of Health Medicine and Caring Science Linköping University, Linköping, Sweden; 4 Pain in Motion Research Group, Department of Physical Therapy, Human Physiology and Anatomy, Faculty of Physical Education & Physical Therapy, Vrije Universiteit, Brussel, Belgium; 5 Department of Health Promoting Science, Sophiahemmet University, Stockholm, Sweden; 6 University of Gothenburg Centre for Person-Centred Care (GPCC), Institute of Health and Care Sciences, Sahlgrenska Academy, Gothenburg, Sweden

**Keywords:** user centered design, digital intervention, pregnancy, support, exercise, prevention, pelvic girdle pain, focus groups

## Abstract

**Background:**

Few pregnant women meet activity guidelines, and about half experience pelvic girdle pain (PGP), with 10% developing chronic symptoms. PGP is multifactorial and tailored physical activity and exercise can reduce pain. A web-based self-care program, Women-In-Motion (WIM), was developed to support physical activity during pregnancy and help prevent and manage PGP.

**Aim:**

The study aim was to explore pregnant women’s experiences with and perceptions of WIM for managing physical activity and PGP to optimize quality of life during pregnancy. A second aim was to use the results for improvements of WIM.

**Method:**

A qualitative approach was employed using focus groups, conducted digitally or in hybrid formats. Pregnant women, gestational weeks <30, were invited. The participants had access to the program one to five weeks prior to focus groups. All sessions were recorded, transcribed, and analysed using a combination of Krueger and Casey’s constant comparison method and Graneheim and Lundman’s inductive content analysis.

**Results:**

Four focus groups were conducted with pregnant women (n=17), representing variation in parity, exercise habits, and experiences of PGP. The analysis resulted in an overarching theme: *“Moving confidently through pregnancy”*. WIM was seen as helping reduce barriers to physical activity while providing biopsychosocial pain insights. Varying support needs indicate more individualized approaches.

**Conclusion:**

Overall, participants experienced the WIM program to enhance their confidence, and their shared perceptions of diverse needs will inform program revisions.

## Introduction

Pregnancy brings significant changes to the female body, with about half of all pregnant women experiencing pelvic girdle pain (PGP).^1,2^ PGP is defined as pain between the posterior iliac crest and gluteal fold, sometimes radiating to the thigh or occurring at the symphysis.^3^ In Sweden, PGP is a leading cause of pregnancy-related sick leave and contributes to significant socioeconomic challenges.^4^ Many pregnant women experience difficulties walking, standing, and rising from a chair, which significantly impacts quality of life.^5–7^ PGP typically resolves within 2–4 months postpartum, but around 25% have troublesome pain one year postpartum^2^ and some women suffer persistent pain for up to 12 years.^8,9^ While hormonal effects on ligament relaxation were once considered a primary cause,^10–12^ this explanation has not been consistently supported in the literature.^13^ Instead, PGP has been associated with several factors across different domains, consistent with the biopsychosocial model of pain, including fear-avoidance of physical activity,^14^ higher BMI (>25),^15^ multiparity,^16^ heavy workload,^17^ stress,^18^ and depression.^19^ The prevailing agreement is that PGP has a multifactorial origin.^20,21^

PGP has a lower incidence among pregnant women who engage in exercise three or more times per week.^22^ When PGP is already present, physical inactivity may worsen symptoms, whereas symptoms can be alleviated with appropriate physical activity and targeted exercises.^23,24^ Physical activity is essential for overall health, particularly in a pregnancy without complications; recommended levels of physical activity are the same as for healthy adults as it lowers the risk for hypertension, diabetes, and depression.^25–32^ However, only a low number (15-27%) of pregnant women meet these physical activity recommendations.^22,33,34^ Pregnancy, particularly the first one, is a critical period when women are open to lifestyle changes,^35^ yet worries about hurting the unborn child and oneself through physical activity may arise^36,37^ and symptoms like PGP, can be obstacles for staying physically active.^6,36,37^ Lack of knowledge about safe activities can contribute to a downward spiral of inactivity, pain, and fear, aligning with the fear-avoidance model.^38,39^ Fear-avoidance beliefs are key predictors of persistent pain, poor outcomes in low back pain (LBP), and persistent PGP.^40–42^

Treatments used to manage PGP in physiotherapy (i.e., individualized exercise, acupuncture, pelvic girdle belts and transcutaneous electrical nerve stimulation) have shown pain reduction and improved functioning with PGP.^24,43–45^ Patient education on biomechanical factors contributing to pain alone can reduce pain in people with LBP and PGP, but should be combined with other physiotherapy treatments such as exercise therapy to yield clinically meaningful improvements.^46^ However, previous studies on patient education have not evaluated pain education with a biopsychosocial perspective or focused on patients’ perceptions of how the information was received and applied.^46^ Although the integration of pain education with a biopsychosocial perspective could potentially lead to further improvements of pain and functioning in the treatment of PGP,^46,47^ this has not been evaluated and represents a gap in the research.

Pain education is an important part of treatment for persistent pain. An increased understanding of pain physiology according to the biopsychosocial model can reduce pain catastrophizing and has shown improved effect on pain and functioning in people with persistent pain.^48^ Avoiding the transition to chronic pain would, however, be even preferable. The current understanding of persistent or chronic pain includes the possibility of nociplastic mechanisms in people with LBP and/or PGP.^49^ Although PGP is typically acute, it carries a risk of developing into persistent and potentially nociplastic pain. To address fear-avoidance behaviours and pain catastrophizing, pregnant women may benefit from pain education and support in adapting physical activity to stay active.

Digital technologies lower the time and requirements of educational expertise or training for the health care provider, which has been seen as a barrier for effective pain education.^50^ While the internet provides valuable information for pregnant women, it can also lead to increased anxiety and greater reliance on healthcare services for support.^51^ This may be mitigated if healthcare providers recommend reliable online resources.

For providing pregnant women information and support for physical activity as well as pain education with a focus on PGP early in pregnancy, we developed the digital self-care program Women-In-Motion (WIM). For a digital self-care program to be effective, it must be engaging, easy to use, and perceived as beneficial.^52^ WIM was developed in collaboration with two pregnant women, one with PGP and one without. The two public research partners were interviewed and performed usability testing, but broader input from women of diverse backgrounds was considered essential to ensure the program meets varied user needs. The aim of this study was to explore pregnant women’s experiences with and perceptions of WIM for managing physical activity and PGP to optimize quality of life during pregnancy. A second aim was to use the results for improving the WIM program before conducting a randomized control trial to evaluate its effectiveness.

## Method

Focus groups discussions (FGD) were used for data collection because they are well-suited for exploring perceptions, experiences, opinions, and behaviours in a collective context. Through discussion, participants can respond to and build on each other’s perspectives, which enriches the data and may reveal shared experiences and differing viewpoints that might not emerge in individual interviews.^53^ FGDs also support innovation development such as in the design and evaluation of the digital intervention WIM.^53^ The sessions were planned to be held on-site with digital delivery as an alternative option.

### Recruitment and participant selection

Participants were recruited according to defined eligibility criteria to ensure that the study sample was relevant to the research aim. Pregnant women, both with and without PGP, were invited personally through contact with midwives or physiotherapists at five primary health care clinics in southwest Sweden. Interested participants scanned a QR-code resulting in a pre-formulated email and were contacted by phone (by BH) to be informed about the study, given the opportunity to ask questions about participation, screened for eligibility and asked background questions (Table 3). Participants received written information by email and gave both written and verbal consent to participate. The participants were provided with WIM login credentials if they agreed to participate.

Inclusion criteria were pregnant women, born in Sweden or abroad, with or without PGP, who were able to read and speak Swedish, and who were enrolled at a midwifery clinic. Women without PGP were included to explore how pregnant women experienced the pain education component, including the information that PGP is a common condition during pregnancy, and whether this led to any concerns or worries.

Exclusion criteria were a history of fracture, malignancy, or surgical interventions of the pelvic floor, pelvis, back, or hip; systemic musculoskeletal or neurological diseases; pregnancy-related diabetes or hypertension; contraindications to intervention; or multifetus pregnancies. The screening process was based on self-reported data and included detailed background questions on participant characteristics. Two participants were excluded (see Supplements). Pelvic health conditions such as symptomatic pelvic organ prolapse or significant birth trauma were not explicitly mentioned in the exclusion criteria and was not screened for if it had not led to surgery.

Within the eligible population, a strategic (purposeful) sampling strategy was employed to optimize the likelihood of including participants who could provide rich and diverse perspectives on pregnant women’s experiences with, and perceptions of, the WIM program. To enhance the diversity of the sample, participants were purposively selected to reflect variation in sociodemographic characteristics (i.e., educational level, cultural background), pregnancy-related factors (i.e., number of pregnancies, presence or absence of PGP), and physical activity levels (Table 3). This approach was intended to maximize the depth and breadth of insights and to enhance the transferability of the findings. After the first two focus groups, all recruiting personnel were instructed to actively invite participants from underrepresented groups.

### The women-in-motion program

Participants had access to WIM between 1-5 weeks prior to the focus groups. The program is accessible via personal login on 1177.se, Sweden’s national public healthcare platform.

The WIM program provides pregnant women with evidence-based information on physical activity and pain management, grounded in findings from previous studies.^1,6,24,28,54–57^ It is a digital self-care program with a smorgasbord design where each user gets access to all information and can choose the components most relevant to them. The three main modules are: physical activity during pregnancy, pain education and management with focus on PGP, and when and where to seek care. The content is delivered through short texts, illustrations and videos. The physical activity content emphasizes its importance during pregnancy, strategies for adaptation, and its role in self-care during PGP. The pain section describes pain physiology and the biopsychosocial factors associated with PGP, highlighting that pain can be managed through multiple approaches. This section has a specific focus on PGP and provides practical tools for management, i.e. overall balance between activity and recovery to home exercise routines and ergonomic advice. For pregnant women experiencing pelvic floor symptoms like urinary incontinence, WIM covers how to adjust physical activity and exercise. WIM employs an active learning approach, with each section featuring reflective questions. In the last part of WIM, the contact information for physiotherapists specialized within women’s health is provided. Participants were advised to contact a healthcare service provider if they experienced increasing or concerning symptoms.

### Procedure

Focus group discussions (FGD) were conducted at the University of Gothenburg between September and December 2024 in a hybrid meeting room with Microsoft Teams used for hybrid and digital meetings. Prior to the first focus group, the semi-structured guiding questions were tested in an individual interview with a pregnant woman. The frameworks for the guiding questions were the biopsychosocial model^58^ combined with pragmatism,^59^ where beliefs are closely linked to actions. The first author (BH) moderated the focus groups, outlining the study’s aim to explore participants’ experiences of the WIM program and its potential for improvement. Each session lasted about 1 hour, and participants were encouraged to engage in open discussions. The participants were informed that the authors were the developers of the WIM-program. After each of the participants had introduced themselves, the moderator started with the guiding question *“What is the first thing that comes to mind when you think of the Women-in-motion program?”*. Additional guiding questions followed depending on the discussion (Table 1). Program images were shown via a PowerPoint-slideshow to support recall. The moderator ensured everyone’s participation and stimulated discussion, while the observers (ML, JK) took fieldnotes and asked clarifying questions. All discussions were recorded with a Dictaphone for verbatim transcription.

**Table 1.** A description of the guiding focus group questions in relation to the theoretical framework.

Conceptual domain	Theoretical/pragmatic basis	Focus group question	Purpose
Overall perceptions and acceptability of the intervention	Pragmatic evaluation of intervention feasibility and user acceptability^59^	What is the first thing that comes to mind when you think of the Women-in-Motion program?	To capture participants’ spontaneous impressions and overall perceptions of the program.
​	​	Would you recommend a friend to use the program? Why or why not?	To explore perceived usefulness, relevance, and acceptability of the intervention.
Understanding of pain within a biopsychosocial framework	Biopsychosocial model of pain emphasizing multiple interacting biological, psychological, and contextual factors^48^	How did you perceive this picture? (illustration of factors increasing/decreasing pain)	To explore participants’ interpretations of the program’s explanation that pain may be influenced by multiple factors.
​	​	How did you interpret the cause of pelvic girdle pain in the program?	To understand how participants interpreted the program’s explanation of pregnancy-related pain.
Experiences with intervention components (physical activity)	Biopsychosocial pain management emphasizing physical activity as a strategy for maintaining function and quality of life^48^	What was it like to use the exercises?	To explore participants’ experiences with the exercise component and its perceived usefulness.
Usability, design, and content of the digital program	Pragmatic user-centered evaluation aimed at improving the intervention before further testing^59^	How did you perceive the content?	To explore whether the information was perceived as understandable and relevant.
​	​	How did you perceive the graphic expression? (animated characters, films, pictures, texts)	To evaluate participants’ experiences with the visual and communicative design of the program.
​	​	What was most annoying about the program?	To identify usability issues and areas for improvement in the intervention.

### Research team and reflexivity

The research team consisted of five physiotherapists (four women, one man). Annelie Gutke (AG), Joanna Kvist (JK), and Mari Lundberg (ML) each have over 30 years’ experience in the field. JK and ML, professors in physiotherapy, primarily focus on research, ML specializing in pain and qualitative methods, including FGD, and JK with experience of research within digital innovations. Jo Nijs (JN) is a professor in physiotherapy and human physiology, and pain researcher. AG, an associate professor combining clinical work with research in women’s health, was responsible for participant recruitment and did not take part in the FGDs. The FGDs were conducted by BH, JK, and ML, none of whom were involved in the clinical care of the participants. BH, is a PhD-student trained in focus group methodology, 17 years of clinical experience as a physiotherapist, including 10 years in primary care. Given BH’s dual role as moderator and main developer of WIM, there was a risk of bias or inhibited critique. This was addressed in advance, and the moderator adopted a reflexive stance demonstrating openness, encouraging all input from participants, and postponing questions about the developer’s thoughts until after the sessions. This reflexive approach acknowledges the researchers’ professional roles and experiences, ensuring transparency in the integration of data while addressing potential influences on the research process.

### Data handling and ethics

The focus group recordings were saved according to the data management plan and after each FGD the moderator and observer reflected on their impressions to keep a good validity and make improvements for the next FGD.^53,60^ As described by Krueger & Casey,^53^ after the first FGD a follow-up question was added to the guiding questions and more pictures were added to the slide-show in order to collect better data. The discussions were transcribed verbatim by BH. All data were coded according to the participant ID given at the inclusion of the study and all files were stored according to the ethical codes and Swedish law. The study received ethical approval from the Swedish Ethical Review Authority (Ref. no. 2020-01575 with addition 2024-01324-02.)

### Data analysis

Recruitment was concluded after the fourth FGD, as no new topics emerged during the audio review. This decision was guided by the principle of data richness in focus group research, a concept similar to saturation.^61^ The FGDs were analysed using an adapted inductive content analysis based on Graneheim and Lundman.^62–64^ This approach was integrated with the constant comparative method described by Krueger and Casey^53^ in order to capture variations and similarities within and between FGD during step two of the analysis (Table 2). The data were analyzed through identification of meaning units, condensation, coding, and abstraction into categories or subthemes and an overarching theme. Analysis was conducted primarily at a manifest level, consistent with the exploratory aim of the study.

**Table 2.** A schematic presentation of the data analysis.

Step	Description
1. A sense of the whole	Each focus group discussion was listened to and read several times to get a sense of their content, and notes were made
2. Grouping data based on study aim	Data were grouped to one health dimension at a time in all focus groups (1. physical activity 2. pelvic girdle pain and 3. quality of life)
2a. Meaning units	The grouped data from the discussions were divided to meaning units
2b. Codes	The meaning units were labelled with a code in a process where the underlying meaning was interpreted
3. Categories/Sub-themes	All codes were compared with each other and codes with similar content were sorted to categories or sub-themes depending on the depth of the material
4. Theme	The categories/sub-themes were condensed into a theme based on the underlying meaning

The focus group methodology is grounded in social constructivism, emphasizing interaction and co-construction of meaning. To honour this perspective, meaning units were kept broad to capture the richness of group discussions. When divergent content was embedded within a broader discussion, the specific sentence was coded separately. NVivo 14 software (Lumivero, USA) was used to facilitate the coding process and the online canvas from Miro (Amsterdam, NL) was used to form the sub-themes.

All analyses were conducted in Swedish to remain close to the original data and preserve the nuances of meaning. Translation into English was done after the analysis was completed, to minimize the risk of losing important content or interpretive depth. Selected citations were translated specifically for inclusion in this manuscript. The participants were not delivered transcripts for correction or analysis for feedback. All content was reviewed and revised by the authors.

### Techniques to achieve trustworthiness and credibility of data analysis

To ensure the trustworthiness and credibility of the data analysis, a preplanned procedure was followed. BH and ML conducted the initial analysis of management of physical activity in the four FGD independently. ML provided methodological support by assisting the selection of appropriate meaning units. Condensation and coding were done independently and compared. In the next step BH did the full analysis for the remaining study aims. Starting at step 3, AG and JK joined the analysis processes and triangulation was conducted by BH, AG, JK and ML. During step 4, JN also joined, and all authors collaboratively refined categories and identified an overarching theme through consensus. The analysis was an iterative process, refined during multiple meetings and manuscript revisions. Credibility was reinforced by representative quotations from the transcribed text to support the findings.^65^ The participants were not delivered transcripts for correction or analysis for feedback. All content was reviewed and revised by the authors.

## Results

Four FGDs were conducted, two in a hybrid format and two fully digital, lasting between 53 and 64 minutes. After attrition (supplement), the groups included four or five participants, with a total of 17 participants (Table 3).

**Table 3.** Participants background characteristics.

Participants	n=17
**Age,** mean (range)	33, (27-39)
**Gestational week**, median (range)	27, (12-33)
**Cultural background** (born and raised/parents)	
Sweden	15
Other country	2
**Children at baseline** (n)	
None	11
One	4
Two	2
**Highest education level** (n)	
Primary school	0
College/Higher Vocational Education	3
University (bachelor/master)	13
PhD	1
**Economy** (self-rated, n)	
Very bad	0
Quite bad	0
Neither good nor bad	4
Quite good	6
Very good	7
**Physical activity over 150 min/week** (self-rated, n)	
Yes	13
No	4
**Exercising 2 times/week or more during pregnancy** (self-reported, n)	
Yes	8
No	9
Pelvic girdle pain* (n)	
Yes	11
No	6

*= assessed with validated questions on phone^66^.

**Figure 1.**
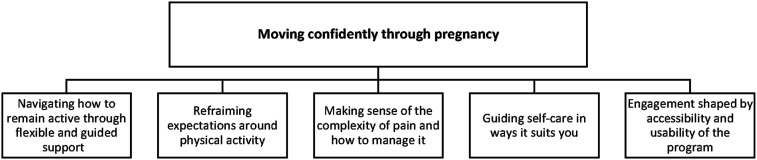
Overarching theme and sub-themes.

Five sub-themes were formed from the coding and resulted in an overarching theme: *Moving confidently through pregnancy*. This captures a central insight from participants’ experiences of WIM. The program was seen as a valuable starting point, lowering the threshold for self-care by providing accessible information and offering recognition and knowledge. Yet participants also highlighted the need for more individualized approaches. Sustainable support for women navigating physical activity and pain in the perinatal period may require a more flexible self-care program than presented to the focus groups participants. As the analysis was conducted at a manifest level, the sub-themes describe different but interrelated aspects of how participants perceived the WIM program. Figure 1.

### Navigating how to remain active through flexible and guided support

Perspectives on the physical activity support provided in WIM varied within and across FGD. Discussions around physical activity was characterized by an initial uncertainty about what was “appropriate” or “safe”, which participants took up and worked through together. Through this interaction, ways of making activity manageable gradually developed, in relation to how WIM presented exercises and reflective questions.

Participants valued the flexibility and adaptability of WIM, which allowed them to engage with exercises and reflective questions at their own pace. While some wished for more detailed guidance on individualized adaptation and ways to increase physical activity, others appreciated the simplicity and clarity of the existing content. A shared view was that the exercise videos were easy to follow, but more guidance on frequency and intensity could help participants tailor the program to their own needs.

As the discussions unfolded, physical activity was talked about as something that could be integrated into everyday life rather than structured exercise aligned with external recommendations. At the same time, uncertainty remained for some participants, particularly how physical activity should be adjusted in later stages of pregnancy, highlighting how flexibility functioned both as a resource and as a point of ambiguity.

**FP2**: *-“But it’s more about, ‘What can I do about it?’ And you might only have 30 minutes to talk about it [in a clinical setting], but here, there are videos you can watch for as long as you need and reflect on. ‘Yes, I can change this.’ ‘I can do this better.’” …*

**Moderator**: -“What do you others say?”

**FP4**: -“Yes, I agree. It was really, really good. You don't have to book an appointment and go away, instead you have it on your own phone or computer or you can take that time and also I like these questions, the ending questions, “think about this”, yes, you can sit and think at home by yourself and take out what you like, what is best for me. I like that.”

This subtheme highlights how flexibility and accessibility in digital self-management can support pregnant women’s engagement in physical activity, while also revealing the need for clearer guidance and more personalized support.

### Reframing expectations around physical activity

Participants consistently described the program’s guidance on physical activity as reassuring and supportive, helping them stay active in ways that felt realistic and achievable. This was particularly valued in contrast to conflicting messages from their social environment, such as being advised to rest, highlighting the importance of clear and supporting messaging. A common experience among participants was an initial feeling of guilt or inadequacy, particularly for those not regularly engaging in exercise or perceiving themselves as unable to meet recommended activity levels. However, the program’s emphasis on all forms of physical activity, including everyday tasks, and on personal adaptation helped mitigate these feelings. A common view among participants was that movement could be tailored to their own routines, abilities, and preferences, reinforcing that there is no single “correct” way to be active.

Another shared view was the need for more specific guidance at certain stages, especially regarding how physical activity affects childbirth and the postpartum period. Participants reflected on previous experiences where guidance and support were limited once the focus shifted to the baby, highlighting variability in informational needs and the value of guidance that is flexible and responsive to personal circumstances.

Overall, the FGDs underscored that participants value support for physical activity that is adaptable, individualized, and sensitive to life stage, allowing them to integrate activity in ways that fit their daily lives.

**FP12:** *…. I think this one is good, yes, as encouragement or motivation regarding physical activity, to sort of say ‘yes’ anyway. It says a lot about the fact that if you haven’t been very physically active, there is still a lot to gain….*

Yes, I think it was good as with that reinforcement.

**Moderator: -Do you others agree, do you also?**

**FP9:** -Yes, I really agree with that. At first you get a little stressed. I'm sitting here watching the contents of the program, and when you read this or watch these videos, why should I exercise, how much and so on do I get a little panicked because you don't live up to it, but there is very good other second-hand information there. That yes, but you shouldn't start with maybe so strenuous exercise if you're not used to it before or if you have some problems and that. You can adapt to the individual and things like that make you feel less pressure than I may be felt first. So, I think that's also very good, maybe just another thing, but.

This subtheme illustrates how supportive and adaptable messaging can help pregnant women reconceptualize physical activity as achievable and personally meaningful, extending beyond structured exercise to movement integrated into everyday life.

### Making sense of the complexity of pain and how to manage it

The FGDs moved from describing isolated physical symptoms of PGP toward a more elaborate understanding in which multiple factors were considered, including sleep, stress, and emotional well-being. This shift was closely linked to how WIM introduced a biopsychosocial perspective. As participants reflected on this perspective, they began to relate their own experiences of PGP to a broader range of influences. This process helped reduce feelings of guilt or the sense of “doing something wrong”. Participants also described that recognizing pain as dynamic and influenced by multiple factors created a sense of hopefulness for change and strengthened their perceived control over their situation. At the same time, information that prior pain experiences may be an influencing factor was reported to cause stress, suggesting the need to tailor WIM content to emphasize modifiable factors.

Factors increasing/decreasing pain were illustrated with a cup containing more or less water. Participants engaged differently with WIM based on their understanding of the analogy. While some described the illustration as intuitive and clarifying, others experienced it as dense or easy to overlook, which in some cases led to disengagement from this part of the content. Overall, participants appreciated the illustration, however they suggested that delivery could be refined to more clearly convey which factors increase the risk of experiencing pain and which additional factors may contribute to pain reduction.

**FP14:** *-Absolutely. As I said, there were many things that you haven't thought of…*

**FP15:** -Yes, but exactly

**FP14:** …if you sleep badly and it can be a factor that is linked to pelvic girdle pain as well. I've never thought about that before. Yes, but also worry.

**FP15 and FP16:** -Mm.

**FP14:** -That it can be such a large part. It's really something that you can sort of work with yourself. So that, absolutely.

This subtheme demonstrates how introducing a biopsychosocial understanding of pain can broaden participants’ perspectives on PGP, fostering greater self-understanding and perceived control, while also highlighting the need for clear and carefully framed communication.

### Guiding self-care in ways it suits you

The role of WIM in relation to professional care was actively negotiated in the discussions as participants drew on their own experiences and responded to each other’s accounts. Participants viewed WIM as a useful resource that could have a larger role in maternal care.

A common pattern among participants was that they had not realized that physical activity could help with PGP or how to adjust movement to stay active, so having this information was appreciated.

WIM was talked about as providing accessible, general guidance that could support self-management of PGP, particularly in situations where participants had previously struggled to find relevant information or navigate care pathways. While a few had received advice from midwives, others had trouble locating physiotherapists with adequate expertise. At the same time, the limits of self-management were made visible through participants’ accounts of previous physiotherapy, which emphasized the importance of personalized assessment and ongoing adjustment. For some, WIM was described as an initial step, while others positioned it as insufficient without parallel professional support. One suggestion was to use WIM before a physiotherapy session. Through these exchanges, digital support and professional care were not treated as interchangeable, but as fulfilling different functions. WIM was positioned in relation to a broader care context, where its usefulness was continuously evaluated against personal needs and experiences.

**FP10:** *I also think that it shouldn’t be a barrier to seek care either before starting the program or alongside it, depending on how one experiences their pelvic girdle pain at the time. I mean, at that specific point in time, one shouldn’t have to try the program first—it should be completely fine to do both.*

**FP9:** *Yes, you're right, and I really agree with that. With a self-management program like this, the responsibility is placed, perhaps rightly so, on the individual. But in my own case, it has been absolutely crucial to get help from this physiotherapist who is incredibly skilled and has been able to give me specific exercises and adjust them each time based on how my body feels. That’s not something you can achieve with a general self-management program. So, for friends and others who experience similar problems, I always say: just get in line to see a qualified physiotherapist. That really is an important point, and I agree with you.*

**FP10:** *Because when you do a program like this, as you say, you don’t have that specialist knowledge yourself. So yes, there’s a limit to how far you can get compared to seeing a professional.*

**FP9:** *So maybe it’s something that needs to be designed in collaboration with the regional healthcare authorities, I don’t know, but to avoid too many people seeking care unnecessarily and to encourage them to use the program instead. But it might also be a reasonable compromise to inform people that they can always seek care in addition to using the program.*

**FP10:** Yes.

This subtheme emphasises that while self-care programs such as WIM can enhance access to support and facilitated self-management, participants still viewed the possibility of professional care as essential for personalized assessment and guidance.

### Engagement shaped by accessibility and usability of the program

Engagement with WIM was discussed in terms of how its accessibility and usability aligned or failed to align with participants’ everyday routines and preferences. Participants across the FGDs generally found the content accessible, highlighting its clear structure and use of diverse formats, including texts, visuals, animations and interactive elements, enabling flexible use and allowing participants to engage with the program at their own pace. At the same time, this flexibility was not experienced uniformly. While some participants described the format as supportive, others disengaged when content was perceived as too text heavy or difficult to navigate. Difficulties in locating exercises or returning to previously viewed content were described as interrupting the flow of use. Several specific improvements were suggested, such as adjusting the AI-generated voice, allowing participants to write responses to reflective questions, adding bookmarking features to easily locate recent content, and making exercises easier to find.

In discussing these experiences, participants compared how they interacted with different parts of the program and what facilitated or hindered continued engagement as ways to make the program more compatible with everyday use. In this way, engagement was made sense of as something shaped by both content and by how the program could be integrated into daily life.

**Moderator:** FP8 and FP5, do you have any thoughts?

**FP8:** No, well in the same way FP7 said - that image I remember I thought “this I can scroll past”. It was too much to read.

**Moderator:** Mm

**FP8:** But it´s very visual. You sort of understand the meaning as soon as you see it, in a way.

**Moderator:** Mm

**FP7:** You understand the concept, sort of.

**Moderator:** Mm

**FP7:** You kind of already have that image in your head, like, it just makes sense without thinking too much

**FP5:** I also thought it was quite clear, but I understand that it can become too much text maybe?

This subtheme underscores that engagement with digital self-care programs depends not only on relevant content, but also on intuitive design, accessibility, and ease of integration into everyday life.

## Discussion

The findings indicate that pregnant women perceived the WIM content as valuable, as it provided new insights into physical activity, pain in general, and PGP in particular. Participants perceived WIM as supportive, reassuring, and clearly presented, containing information which should be given a more substantial role in maternal care. The interpretation of the overall experience was that it contributed to a sense of confidence. For PGP this was grounded in the findings forming the sub-theme *Making sense of the complexity of pain and how to manage it*. Our results are in line with earlier studies investigating the needs of pregnant women, which highlights the need for more information on what PGP is, how it can be managed, and advice on how to cope with the symptoms.^67,68^ One of the studies highlight information and communication technologies as resources that might be able to promote self-care.^67^

According to the sub-theme *Reframing expectations around physical activity* the content was reassuring and supportive, helping the pregnant women to stay active in ways that felt realistic and achievable. Our goal is that the use of WIM can improve the number of pregnant women achieving the recommended physical activity levels by lowering the threshold for what is needed and how to do it.

Overall, the analysis highlights women’s varying needs, emphasizing the importance of delivering a digital self-care program with options for individualization. While the WIM program uses a smorgasbord approach to support individualization by allowing users to engage with content that suits their needs and preferences, focus group participants were instructed to complete the entire program for feedback purposes, which may have made it seem less flexible than it truly is. However, the results indicate that pregnant women have diverse needs regarding information delivery, types of support, and even specific elements such as the number of exercises provided. This aligns with studies on eHealth app development, underscoring the need for more individualized care.^69,70^ WIM may also have the potential to address this need.

Discussions forming the sub-theme *Guiding self-care in ways it suits you* reflected the participants appreciation for personalized care and the challenge of accessing it. Notably, the last part of WIM aims to facilitate individual contact with relevant healthcare professionals, with contact information to specialized physiotherapists, for those who need it. Encouraging self-care before contacting a physiotherapist is intended to promote personal responsibility, but it should not limit access to support when needed. This creates an ethical dilemma, as such guidance may be interpreted in different ways: some individuals may feel obliged to persist on their own, while others may choose to disregard it. As a result, providing the same information can lead to unequal outcomes, thereby raising concerns about fairness, autonomy, and the risk of unintended burden.

Within the sub-theme *Navigating how to remain active through flexible and guided support* the need for additional support to become more physically active was highlighted, and this was interpreted by the researchers as a call for the use of more behavioural change techniques (BCTs).^71^ The WIM program already includes certain BCTs, like problem solving and action planning, through the reflective questions. BCTs such as goal setting, goal review, and feedback, may support increased physical activity among pregnant women.^72^ However, motivational counselling appears less effective than supervised exercise, raising questions about the practical relevance of BCTs in this context.^73^ Further research is needed to clarify which support strategies are most effective to promote physical activity in pregnant women. In the present study, the target population is pregnant women, both physically active and inactive, with the goal of supporting physical activity while preventing PGP or minimizing its symptoms. One finding that sheds light on this diverse population concerned the perceived need for exercises: more physically active women wanted additional content, while less active participants expressed concern that adding more might feel overwhelming. It should be noted, however, that the proportion of physically active participants in this study was markedly higher than that reported in observational studies of pregnant women.^33,34^ Results should therefore be interpreted with caution, ensuring that WIM addresses a wide range of preferences of physical activity. Perceived differences in desired support from the digital program highlights the need for research on how healthcare providers can personalize maternity care within limited resources.

In the sub-theme *Engagement shaped by accessibility and usability of the program* participants appreciated short videos and varied content delivery but perceived one illustration (factors increasing and decreasing pain) in WIM as overly complex and unclear, limiting engagement. This highlights the need to adapt educational content to diverse user backgrounds and literacy levels. To enhance usability, the pedagogical presentation should be revised to improve clarity and emotional engagement, in line with approaches outlined by Moseley et al.^74^ regarding the development of pain science education, its learning frameworks, and delivery strategies.

As the purpose of the study was to explore variation in perceptions rather than to attribute patterns to predefined participant variables, participant background characteristics were used to describe the sample and support assessment of transferability, and not to conduct explanatory subgroup analyses. Although all background characteristics were represented except for low income, only a few participants had lower educational levels or a different cultural background. This may have influenced the findings by excluding important perspectives and must be acknowledged as a limitation of the study. Efforts to increase representation from underrepresented groups (lower education and diverse cultural backgrounds) were unsuccessful. Four participants with lower educational attainment, of which three had other cultural backgrounds, withdrew after inclusion. Future studies should consider how information is delivered, the location of FGDs, offering support for digital participation and the use of incentives for low-income participants, as these aspects were reported as barriers.

Providing technical support for login to the self-care program may also be important. Two participants who withdrew had not logged in to WIM and could not be reached by phone before withdrawal, whereas one woman with lower education and a different cultural background participated after receiving telephone guidance.

Although the sessions were originally planned to be held on site, digital participation was also an option. The study comprised two fully digital and two hybrid FGDs. A number of participants preferred digital participation, and flexible participation options have previously been shown to be important.^75^ Digital formats have not been shown to reduce willingness to share sensitive experiences,^76^ or affect outcomes^77^ and can be less time-consuming.^78^ To our knowledge, hybrid FGDs are uncommon. Earlier findings from digital FGDs have found issues regarding nonverbal interactions and taking turns.^79^ In this study we did not experience this as an obstacle, possibly due to the small number of participants in each group and the information to digital participants to have their microphone and video on during the whole discussion. Nevertheless, the hybrid format may warrant further research as it has not, to our knowledge, been evaluated.

In this study, we used an adapted content analysis using broad meaning units that reflected the discussions, combined with the constant comparative method often used within focus group analysis. Graneheim, Lindgren, and Lundman describe the analytic process as: *“No method is absolutely weak or strong, just more or less useful in relation to a certain aim”*,^64^ a view supported by Krueger & Casey.^53^ The combination of methods was considered by the authors to be a well-balanced adaptation of the analytical approach to suit the study’s aim.

## Conclusion

The findings indicate that pregnant women value structured support for physical activity and pain management with a biopsychosocial perspective, which is currently lacking in routine maternity care. WIM addresses this gap by offering digital guidance and evidence-based information. This study of WIM expands current knowledge regarding how desired support and opportunity for self-care are perceived as leading to confidence and are well received by the intended user group.

The effectiveness of WIM in promoting physical activity and supporting pain management will be evaluated against standard care in an RCT. If positive effects are demonstrated, WIM can be implemented early in maternity care.

## Supplemental material

Supplemental material - Pregnant women’s experiences of the digital self-care program *women-in-motion* to manage physical activity and pelvic girdle pain: A qualitative study Supplemental material for Pregnant women’s experiences of the digital self-care program women-in-motion to manage physical activity and pelvic girdle pain: A qualitative study by Bodil Halvarsson, Annelie Gutke, Joanna Kvist, Jo Nijs, and Mari Lundberg in Digital Health.

Supplemental material - Pregnant women’s experiences of the digital self-care program *women-in-motion* to manage physical activity and pelvic girdle pain: A qualitative study Supplemental material for Pregnant women’s experiences of the digital self-care program women-in-motion to manage physical activity and pelvic girdle pain: A qualitative study by Bodil Halvarsson, Annelie Gutke, Joanna Kvist, Jo Nijs, and Mari Lundberg in Digital Health.

## Data Availability

The datasets generated and/or analysed during the current study are not publicly available due individual privacy but are available from the corresponding author on reasonable request.*

## Appendix

### List of abbreviations

PGP: Pelvic girdle pain
WIM: Women-In-Motion, the digital self-care program
FGD: Focus group discussions.
