# Supplementary material for: Pregnant women’s experiences of the digital self-care program women-in-motion to manage physical activity and pelvic girdle pain: A qualitative study
Source: Digit Health. 2026 Jun 9;12:20552076261459519. doi: 10.1177/20552076261459519 (PMC13250426; doi:10.1177/20552076261459519)
Supplement: Supplemental material - Pregnant women’s experiences of the digital self-care program women-in-motion to manage physical activity and pelvic girdle pain: A qualitative study [file sj-pdf-1-dhj-10.1177_20552076261459519.pdf]

Meaning Unit	Condensed MU	Code	Category
**FP10:** I think I agree with FP12 that videos can be really boring so that you. Yeah, now we perhaps had a reason to go through all four parts [of WIM], but it´s really individual, how you, so to say, what you need help for. Some have a lot of PGP and want to focus on that and read that first while others who don´t have pain yet maybe want well…, how to prevent it and go directly to the physical activity part. And not like this -This is how you should do it. But instead that you are free to choose what to start with from the beginning, and then that you can jump to other parts as well, yes.<br><br>**FP11:** But I think that's a little tricky, because now as I am in this study, I really wanted to look at everything, but otherwise I would have also jumped around a little. But I think that´s unfortunately afterwards, Yes, I think it was good to do it in the right order or so, because it emerged [new] things in the parts where I thought, this I know, or understand. So, in a way I thought it was good to do it in the right order, at the same time, it is good if one is going to have this as an app or something, that it should be easy to use.<br>Then you need to be able to choose or return to something that you want to listen to again or so.	It´s important to be free to choose, but it was good to go through it in the right order	Easy to understand with short videos and the possibility to choose the order	Structural and communicative design features that influence effective utilization

**FP17:** There was still quite a lot of information and advice you could make use of on your own, things I didn't know about [before]. I mean, sure, you've heard that putting a pillow between your legs is supposed to be more comfortable, but apart from that, at least I didn't really have much knowledge. So it felt like there actually was a lot you could do, before [seeking care].<br><br>**Moderator:** mm<br><br>**FP17**: But then, yes, when you have tried things, then you know. Well, if it doesn´t get better now, then maybe I need to seek care anyway.<br><br>**Moderator:** Do you feel informed or secure with [that information] when do I need to seek further care?<br><br>**FP14:** That´s always hard, I think, when it comes to something completely new, when you experience new pain and emotions and thoughts and you don´t know if you should process [them]. But it gave you a little more, sort of -OK, maybe I should try this?	There is a lot to do before you seek [care] that I did not know about. New feelings are hard [to interpret] but it [WIM] gave a little more [advice] what you can try out	There is a lot you can do on your own, but even if you get good instructions, you can need individualized care from specialized physios.	Self-care information is valuable but individualized support may be required
