# Supplementary figures and images for: Pregnant women’s experiences of the digital self-care program women-in-motion to manage physical activity and pelvic girdle pain: A qualitative study
Source: Digit Health. 2026 Jun 9;12:20552076261459519. doi: 10.1177/20552076261459519 (PMC13250426; doi:10.1177/20552076261459519)

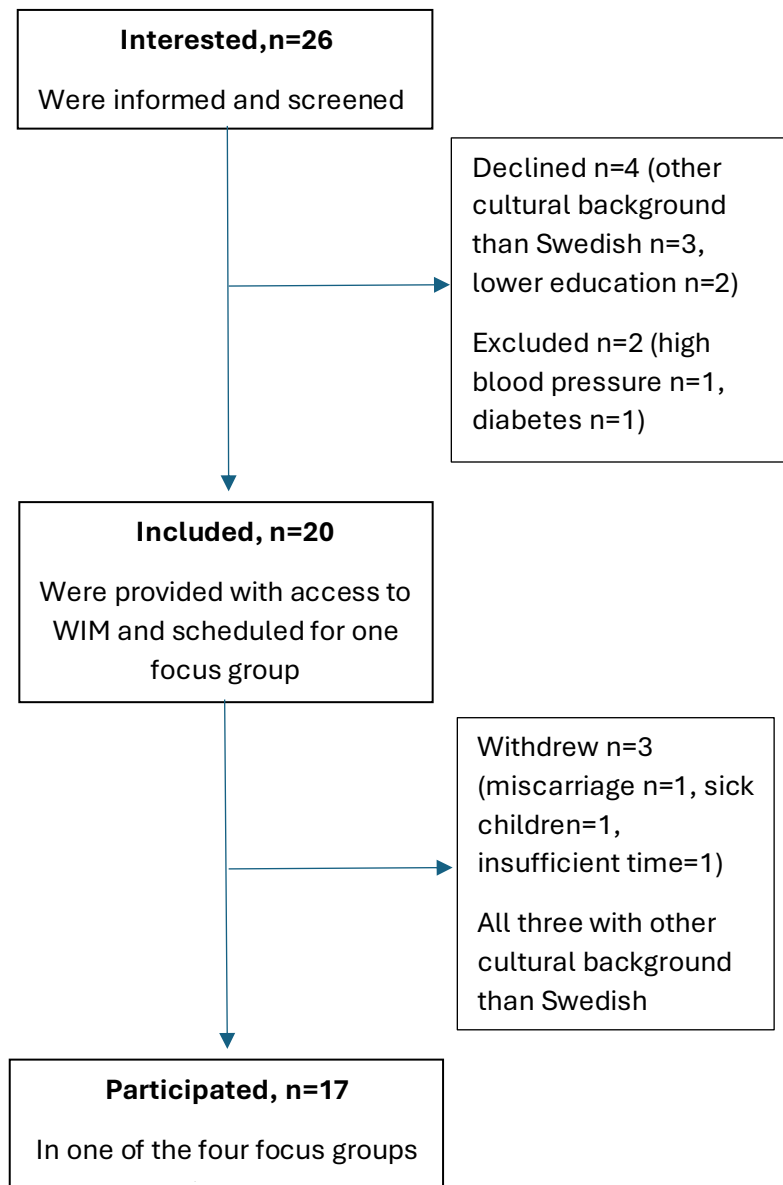

Figure. 2 Flowchart of study participants

Supplement: Supplemental material - Pregnant women’s experiences of the digital self-care program women-in-motion to manage physical activity and pelvic girdle pain: A qualitative study [file sj-pdf-2-dhj-10.1177_20552076261459519.pdf]
